# Non-functioning pancreatic neuroendocrine tumor presenting with acute pancreatitis: a case report

**DOI:** 10.1093/omcr/omae207

**Published:** 2025-03-20

**Authors:** Aref Al-Rajabi, Amal A Thweib, Natalia M Daghlis, Howaida A Rabba, Nameer AbuMunshar, Abdel Majeed Al Ali, Izzeddin A Bakri

**Affiliations:** Assistant Professor, Palestine Polytechnic University; Al Ahli Hospital, Gastroenterology, West Bank, Hebron P700, Palestine; College of Medicine and Health Sciences, Palestine Polytechnic University, West Bank, Bethlehem P150, Palestine; College of Medicine and Health Sciences, Palestine Polytechnic University, West Bank, Salfeet P390, Palestine; Collage of Medicine and Health Sciences, Palestine Polytechnic University, West Bank, Hebron P700, Palestine; Collage of Medicine and Health Sciences, Palestine Polytechnic University, West Bank, Hebron P700, Palestine; Gastroenterology, Al Ahli Hospital; Najah National University Hospital, Nablus, West Bank, Hebron P700, Palestine; Pathology Department, Al Ahli Hospital, West Bank, Hebron P700 Palestine

**Keywords:** gastroenterology, radiology

## Abstract

Pancreatic neuroendocrine tumors (pNETs) are a group of tumors with complex therapeutic options that differ according to pathological grading, clinical staging, and the existence of hormone secretion symptoms. We report a 34-year-old male with recurrent acute pancreatitis due to a non-functioning pNET. He presented with intermittent epigastric pain and elevated pancreatic enzymes. MRCP and endoscopic ultrasound revealed an oval lesion near the pancreatic head, confirmed as a grade 1 neuroendocrine tumor on biopsy. A Whipple procedure was performed based on tumor size and location. This case highlights the rare occurrence of pNET presenting with acute pancreatitis.

## Introduction

Pancreatic neuroendocrine tumor (pNET) represents fewer than 2% of all pancreatic cancer cases and is a collection of uncommon epithelial neoplasms [1]. The pNET has a median age of less than 50 years [2] and an equal male-to-female incidence, affecting approximately 1.5 out of every 100 000 people annually [3].

pNETs are classified as either functioning or non-functioning. Functioning tumors are typically diagnosed early due to associated endocrine symptoms, while non-functioning tumors often remain asymptomatic until they reach an advanced stage or metastasize. In rare cases, non-functioning pNETs may present with acute pancreatitis, potentially due to pancreatic duct obstruction by mass or ischemia from vascular obstruction [4].

We present a case of a patient with acute pancreatitis secondary to PNT, along with a literature review.

## Case presentation

A 34-year-old male patient with no family history of cancer has a history of cholelithiasis. A year ago, he underwent cholecystectomy and has experienced recurrent episodes of pancreatitis; the first episode of pancreatitis occurred five months before the diagnosis of cholelithiasis and was treated conservatively. The second episode occurred three months later. In this episode, lipase was 1466, and amylase was 388; the patient was admitted to the ICU as an NPO case for three days until the infection subsided. An MRCP showed an oval-shaped soft tissue-like lesion measuring approximately 26 mm × 18 mm × 28 mm, located posterior and inferior to the pancreatic head, exerting mass effect upon the IVC. The patient presented to the hospital with a third episode of intermittent epigastric pain lasting five days, with a gradual onset and no history of vomiting or weight loss.

The vital signs were stable. There was no jaundice or palpable lymph nodes; the abdomen was soft and lax with no tenderness, and no palpable mass or organomegaly. Otherwise, the examinations were normal, with SGPT at 81 uU/ml.

**Figure 1:**
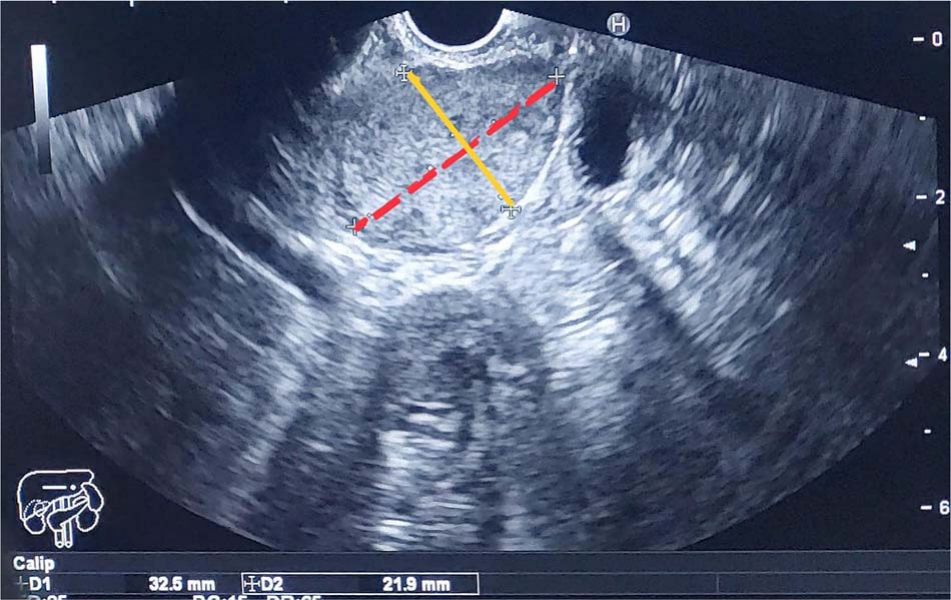
The image shows the two dimensions of the mass as measured by EUS: The first dimension, represented by a dashed line, measures 32.5 mm, and the second dimension, indicated by a solid line, measures 21.9 mm.

A multiplane MRCP was performed as a follow-up, which showed a progressive oval-shaped soft tissue lesion measuring approximately 29 mm × 19 mm × 32 mm. The patient underwent endoscopic ultrasound (EUS), fine-needle biopsy (FNB), and fine-needle aspiration (FNA). The EUS revealed a large, rounded heterogeneous lesion about 30 mm × 30 mm in size, inferior to the head of the pancreas, pushing the duodenal wall and ampulla (Fig. 1).

**Figure 2:**
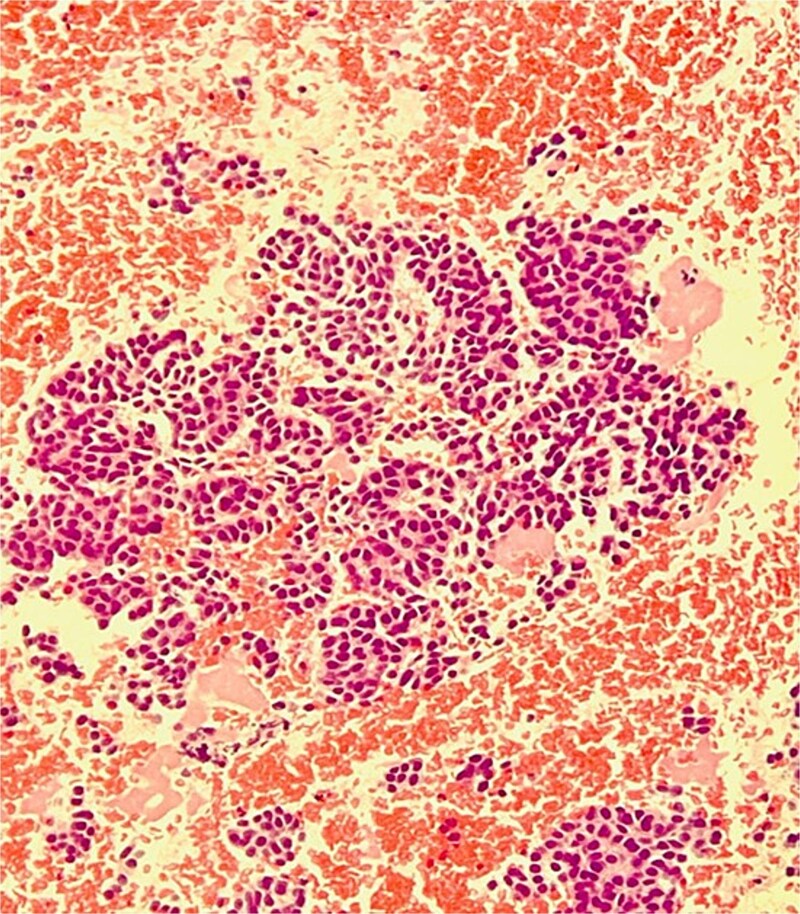
The image shows small to medium sized cells with eosinophilic to amphophilic and finely granular cytoplasm arranged in nests and tubuloacinar architecture.

**Figure 3:**
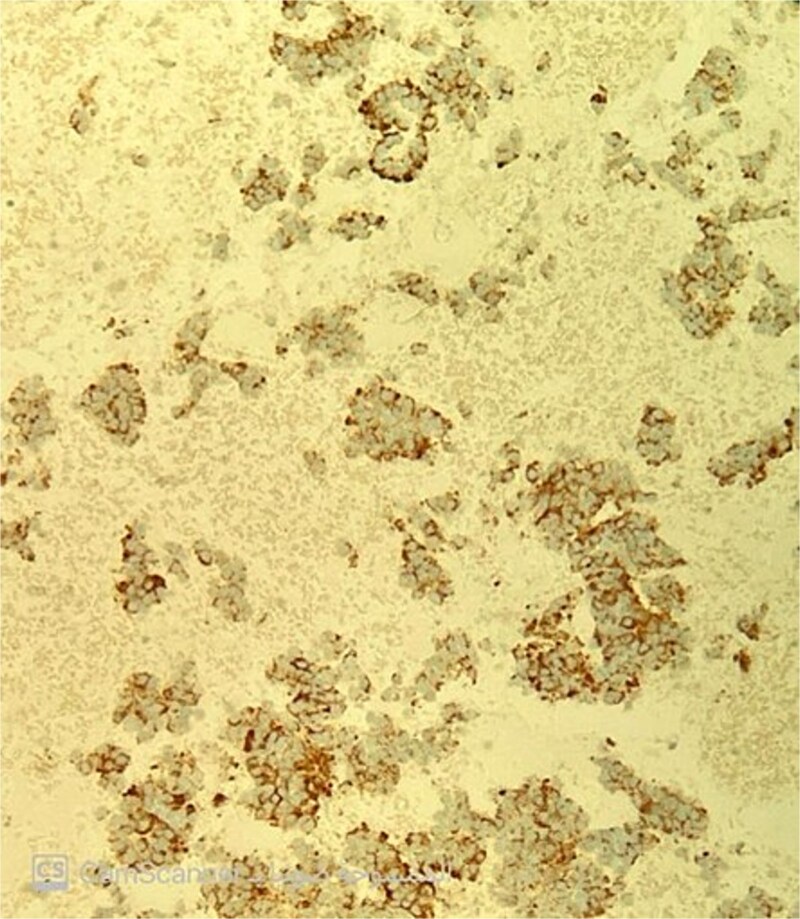
Chromogranin immunostain is positive.

The pathology report confirmed a well-differentiated neuroendocrine tumor. Hematoxylin and eosin staining revealed small to medium-sized cells with eosinophilic to amphophilic, finely granular cytoplasm arranged in nests and tubuloacinar architecture (Fig. 2). Immunohistochemistry showed positivity for chromogranin (Fig. 3) and pan-cytokeratin, while desmin and EMA markers were negative. The Ki-67 proliferation index was less than 2%, consistent with a grade 1 (low-grade) tumor.

Based on the pathology results, the patient underwent the Whipple procedure. The Whipple specimen shows a mass measuring 3 cm × 2 cm × 2 cm, limited to the pancreas (T2), with lymphatic invasion and perineural invasion present. A total of nine lymph nodes were retrieved, two of which were positive for malignancy.

## Discussion

Neuroendocrine neoplasms (NENs) are a class of tumors arising from neuroendocrine cells that are present in all organs, pancreas being the most common location. pNETs are rare tumors with less than or equal to one case per 100 000 people annually [5], that make up less than 2% of all pancreatic neoplasms. They are WHO classified into grades according to morphological differentiation and mitotic labeling index ki-67.

Grade 1 pNET a well differentiated tumor or low-grade tumor with mitotic rate < 2 mitoses/2 mm^2^ and Ki-67 index < 3%, Grade 2 pNET a well differentiated tumor or intermediate grade tumor with mitotic rate 2–20 mitoses/2 mm^2^ and Ki-67 index 3–20%, Grade 3 pNET a well differentiated tumor or high-grade tumor with mitotic rate > 20 mitoses/2 mm^2^ and Ki-67 index > 20% [6].

There is a well-established association between exocrine pancreatic cancer and both acute and chronic pancreatitis. This relationship arises because exocrine pancreatic cancer often causes blockage of the major pancreatic duct due to tumors within the ductal system. In the current case, the blockage of the ductal system by the pNET is the main cause of recurrent pancreatitis [7]. In reviewing the literature, 23 cases of acute pancreatitis (AP) in patients with pancreatic neuroendocrine tumors (pNETs), most of which were non-functional types with mild AP episodes. This case report adds to the rare documentation of non-functional pNETs presenting with recurrent AP [4].

The primary causes of acute pancreatitis (AP) are gallstones and alcohol abuse, both of which were ruled out in this patient based on history and investigations. Less common causes include trauma, viral infection, and exocrine pancreatic tumors [4].

Accurate diagnosis and treatment depend on the tumor’s progression and location. MRI and CT scan results lack high specificity and sensitivity; however, accuracy can be improved by using helical CT in conjunction with PET scans. Endoscopic ultrasound (EUS) is the most effective method for determining the tumor’s site, size, and extent [8].

Liver metastasis significantly impacts disease progression, with a 5-year survival rate of 80% in the absence of liver metastasis, compared to 50% in the presence of diffuse liver metastases [9].

We describe a case of a progressive lesion in the head of the pancreas in a patient with a low-grade non-functioning pNET, characterized by recurrent acute pancreatitis, identified by MRCP and confirmed by EUS-FNA. The patient had no family history of pancreatic tumors and denied alcohol intake. The treatment of choice for patients with well-differentiated, localized tumors greater than 2 cm and no metastasis is Whipple surgery or pancreaticoduodenectomy [10].

For the first three years after surgical resection, follow-up consultations with cross-sectional imaging (triple-phase CT or MRI) should occur annually. After that, imaging should be conducted every one to two years for a total of ten years, along with routine monitoring for recurrences using serum biomarkers, including pertinent hormones [5].

Treatment for pancreatitis varies by severity. For mild acute pancreatitis, management includes pain relief, resuscitation, and a regular diet. In severe cases, patients require admission for monitoring and enteral feeding. Prophylactic antibiotics are only recommended for necrotizing pancreatitis. For acute gallstone pancreatitis, ERCP is considered, and if not feasible, percutaneous transhepatic gallbladder drainage is an option.

For unresectable Grade 1 or 2 pNETs without liver metastases, treatment options include somatostatin analogs and peptide receptor radionuclide therapy. If liver metastases are present, resection or selective internal radiotherapy may be considered.

For unresectable Grade 3 pNETs, streptozocin and 5-fluorouracil are preferred, while NEC is typically treated with cisplatin-based chemotherapy and etoposide.

## Conclusion

In this case, we discuss the relationship between pancreatitis and neuroendocrine tumors.

Even in people under 50, pancreatic neuroendocrine tumors can result in acute pancreatitis. Acute pancreatitis may be the initial clinical symptom in some cases because the tumors are not functioning.

## Consent

Written informed consent was obtained from the patient for publication of this case report and accompanying images.
